# Scanning tunneling spectroscopy of epitaxial graphene nanoisland on Ir(111)

**DOI:** 10.1186/1556-276X-7-255

**Published:** 2012-05-15

**Authors:** Soo-hyon Phark, Jérôme Borme, Augusto León Vanegas, Marco Corbetta, Dirk Sander, Jürgen Kirschner

**Affiliations:** 1 Max-Planck-Institut für Mikrostrukturphysik, Weinberg 2, Halle, 06120, Germany; 2 International Iberian Nanotechnology Laboratory, Avenida Mestre José Veiga,, Braga, 4715-310, Portugal

## Abstract

Scanning tunneling spectroscopy (STS) was used to measure local differential conductance (d*I*/d*V*) spectra on nanometer-size graphene islands on an Ir(111) surface. Energy resolved d*I*/d*V* maps clearly show a spatial modulation, which we ascribe to a modulated local density of states due to quantum confinement. STS near graphene edges indicates a position dependence of the d*I*/d*V* signals, which suggests a reduced density of states near the edges of graphene islands on Ir(111).

## Background

Graphene (G) has attracted significant attention because of its special and unique physical properties which make it a promising future material for nanoelectronics [1]. In nanosize G structures, confinement geometry and increased edge-to-area ratio are expected to influence the electronic properties of G significantly. Previous studies showed how confinement of carriers in a G nanostructure affects the electrical conductivity [2] and the energy gap [3]. Edge-localized electrons near the zigzag edge of a freestanding G nanoribbon were proposed by Nakada et al. [4]. Recently, C. Tao et al. [5] reported electronic edge states dependent on the edge atomic orientations in G nanoribbons.

A theoretical study [6] predicted a small binding energy of *≈* 50 meV per C atom in G/Ir(111). G *π*-band related features were observed in angle-resolved photoemission spectroscopy studies of G/Ir(111) [7]. However, a strong edge-substrate interaction in G nanoislands on Ir(111) has been proposed [8]. This motivates a spatially resolved investigation of spectroscopic features of G/Ir(111) nanostructures to elucidate the spatial dependent electronic properties and the influence of the substrate on the electronic states near a G edge, and corresponding results are presented here.

We present scanning tunneling microscopy (STM) and scanning tunneling spectroscopy (STS) measurements on G nanoislands on Ir(111). The d*I*/d*V* spectra and maps show a spatial modulation, indicative of a modulated local density of states (DOS), which is ascribed to electronic quantum confinement [9]. STS near the edge of a G island on Ir(111) shows a reduction of the differential conductance d*I*/d*V*. This reflects a change of the G electronic structure, which we ascribe to the different G-Ir interaction near the edge as compared to the central region of the G island.

## Methods

Monolayer G islands were grown by exposing a clean and atomically flat Ir(111) surface to C_2_H_4_ at 300 K at a pressure of 2 *×* 10^*−*9^ mbar, followed by subsequent heating of the substrate to 1,320 K. STM and STS measurements were performed at 8 K [9]. Constant current (CC)-STM images showed that the island diameter ranges from 6 to 50 nm. We employed a lock-in technique with a modulation bias voltage at a frequency *ν* = 4 kHz and root-mean square amplitude of 20 mV to detect *I*(*V*) and d*I*/d*V* simultaneously.

## Results and discussion

**Figure 1:**
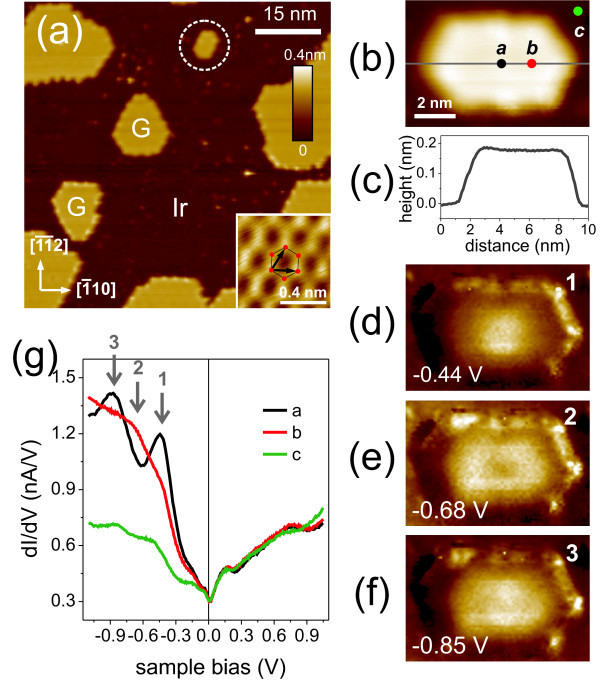
**STS on a graphene nanoisland on Ir(111).** (**a**) A 70 *×* 70 nm^2^ CC-STM image of G islands on Ir(111) (*V*_*S*_ = *−*0.05 V, *I*_set_ = 1 nA). The inset is a CC-STM image of a zoom-in of the G lattice with an illustration of the hexagonal C ring (red circles) and lattice vectors (black arrows) (*V*_*S*_ = 0.05 V, *I*_set_ = 1 nA). Crystallographic directions of the Ir substrate are denoted at the bottom-left side, as deduced from atomically resolved STM images of the substrate. (**b**) The G island indicated by the dashed circle in (a). (**c**) It gives the line profile along the long axis of the island in (b). (**d,e,f**) The d*I*/d*V* maps of the island in (b), measured at *V*_*S*_ denoted at the bottom-left corner of each image. (**g**) STS spectra measured at the positions *a* to *c* in (b).

Figure 1a,b shows G islands grown on an Ir(111) surface and a nanoisland used for a STS measurement, respectively. The CC-STM image measured on the central part of the G island shows a clear honeycomb structure as shown in the inset of Figure 1a. The line profile of the island reveals an apparent height of approximately 0.2 nm, as shown in Figure 1c, which identifies the island as single layer G. Figure 1g shows d*I*/d*V* curves measured at different positions of the island shown in Figure 1b. The curve measured at position *a* (the center of the island) shows two prominent peaks at a sample bias *V*_*S*_ *≈ −*0.44 and *−*0.85 V. However, the curve measured at position *b* shows a smaller d*I*/d*V* signal at those *Vs* values and a larger signal at an intermediate *V*_*S*_. These *V*_*S*_-dependent and spatially modulated d*I*/d*V* signals are ascribed to electron confinement, which induces a corresponding spatial modulation of local DOS [9-11]. This can qualitatively be understood by the inspection of the energy-dependent d*I*/d*V* maps.

We measured the d*I*/d*V* maps of the G island in Figure 1b as a function of *V*_*S*_. Figure 1d,e,f shows the d*I*/d*V* maps at *V*_*S*_ denoted by the gray arrows in Figure 1g. As we lower *V*_*S*_ from zero bias, the first modulation pattern (Figure 1d) is observed at *V*_*S*_ *≈ −*0.44 V of peak ‘1’ of the d*I*/d*V* curve measured at position *a*. Here, the d*I*/d*V* signal shows a maximum at the center of the island and a monotonic decrease towards the edges. For larger negative *V*_*S*_, the maps at two other *V*_*S*_ values (peaks ‘2’ and ‘3’) show different spatial distributions of the d*I*/d*V* signals as compared with that of the first one. These results can be understood as standing wave patterns which resulted from the interference of electron waves. The energy-dependent variation of the spatially modulated d*I*/d*V* signal suggests a variation of the electron wavelength as a function of electron energy in the G nanoisland. Thus, the position-dependent STS spectra and spatially modulated d*I*/d*V* signals provide a clear evidence for electron confinement in the G nanoisland. The energy eigenvalues of the confined electron states are determined by the geometry of the confinement potential (the size and shape of the island). This is also true for the graphene nanoislands [12]. We observed a different onset energy of the lowest quantum-confined state (the first peak) in other graphene islands with different shape and size. We performed an extensive quantitative analysis of modulation patterns on G islands and extracted the electron dispersion relation [9].

**Figure 2:**
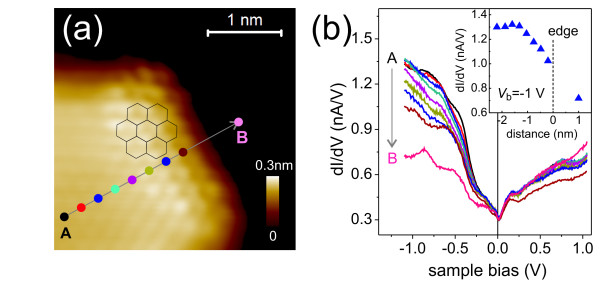
**STS near an edge of graphene nanoisland.** (**a**) Atomic resolution CC-STM image of top-left side of the island in Figure 1b (*V*_*S*_ = *−*0.05 V, *I*_set_ = 1 nA). The hexagons denote the honeycomb lattice structure of G. (**b**) STS spectra measured at nine positions along the line *AB* across the G edge towards the Ir region. The inset shows the d*I*/d*V* signal height at *V*_*S*_ = *−*1.0 V as a function of distance from the edge. The d*I*/d*V* signal decreases from the position of approximately 1.0 nm inside the island towards the edge.

We also performed spatially resolved STS measurements near G edges. Figure 2a is an atomically resolved CC-STM image of the top-left region of the island of Figure 1b. Figure 2b shows the d*I*/d*V* spectra measured along different positions from G towards Ir. For negative *V*_*S*_, the d*I*/d*V* signal of G is larger than that of Ir(111) by a factor of 2. However, the overall similarity of the d*I*/d*V* signals of Ir and G/Ir is striking. We may ascribe this to the electronic hybridization between G and Ir [6]. The inset of Figure 2b shows the d*I*/d*V* signals at *V*_*S*_ = −1.0 V as a function of positions from A to B in Figure 2a. As the measurement positions move from G to Ir, we find a monotonic decrease of the d*I/*d*V* signal without the appearance of additional features within the distance of approximately 1 nm from the edge. We do not observe peculiar edge-related states, which were discussed for other systems [5] or have been described in theory [4].

Lacovig et al. [8] discussed, based on *ab initio* calculations, that the interaction between a G edge and Ir is strong enough to induce a considerable reduction (approximately 50%) of the C-substrate distance near the edges as compared with that of the central region of a G island on Ir(111). This structural relaxation should also impact the electronic DOS near an edge as compared to the central region of an island. The reduction of the d*I*/d*V* signal in Figure 2, which can be attributed to the reduction of the G DOS at the edges, possibly reflects this. We speculate that an unsaturated *σ* orbital at the edge tends to bond to the Ir atoms with a covalent character, which would reduce the edge energy and deplete the DOS near *E*_*F*_. This could explain the decrease of d*I*/d*V* signals in Figure 2. In addition, the electronic relaxation due to a C-Ir interaction at the edge could be the reason why our STS measurements do not show peculiar electronic states in the edge region, in contrast to the prediction of theory for the edge of freestanding G [4].

## Conclusions

We investigated electronic properties of G nanoislands on Ir(111) by STM and STS. The d*I*/d*V* spectra and maps show a pronounced spatial modulation, indicative of a modulated local DOS. We ascribe this to a quantum confinement of electrons in G nanoislands. We also performed STS near the edge of a G island on Ir(111). Spatially resolved tunnel spectroscopy indicates a considerably reduced density of states. This reflects a change of the G electronic structure, which we ascribe to the different G-Ir interaction near the edge as compared to the central region of the G island.

## Competing interests

The authors declare that they have no competing interests.

## Authors' contributions

S-HP, DS, and JK conceived and designed the experiments. S-HP, JB, ALV, and MC prepared samples and performed STM and STS measurements. S-HP, JB, and ALV analyzed STM and STS data. All authors discussed the results and wrote the paper. All authors read and approved the final manuscript.

## References

[B1] Castro Neto AH Guinea F Peres NMR Novoselov KS Geim AK The electronic properties of graphene Rev Mod Phys 2009 81 109 162 10.1103/RevModPhys.81.109

[B2] Berger C Song Z Li X Wu X Brown N Naud C Mayou D Li T Hass J Marchenkov AN Conrad EH First PN de Heer WA Electronic confinement and coherence in patterned epitaxial graphene Science 2006 312 1191 1196 10.1126/science.1125925 16614173

[B3] Ritter KA Lyding JW The influence of edge structure on the electronic properties of graphene quantum dots and nanoribbons Nat Mater 2009 8 235 242 10.1038/nmat2378 19219032

[B4] Nakada K Fujita M Dresselhaus G Dresselhaus MS Edge state in graphene ribbons: nanometer size effect and edge shape dependence Phys Rev B 1996 54 17954 17961 10.1103/PhysRevB.54.17954 9985930

[B5] Tao C Jiao L Yazyev OV Chen YZ Feng J Zhang X Capaz RB Tour JM Zettl A Louie SG Dai H Crommie MF Spatially resolving edge states of chiral graphene nanoribbons Nat Phys 2011 7 616 620 10.1038/nphys1991

[B6] Busse C Lazić P Djemour R Coraux J Gerber T Atodiresei N Caciuc V Brako R N’Diaye AT Blügel S Zegenhagen J Michely T Graphene on Ir(111): physisorption with chemical modulation Phys Rev Lett 2011 107 036101 21838377 10.1103/PhysRevLett.107.036101

[B7] Kralj M Pletikosić I Petrović M Pervan P Milun M N’Diaye AT Busse C Michely T Fujii J Vobornik I Graphene on Ir(111) characterized by angle-resolved photoemission Phys Rev B 2011 84 075427

[B8] Lacovig P Pozzo M Alfé D Vilmercati P Baraldi A Lizzit S Growth of dome-shaped carbon nanoislands on Ir(111): the intermediate between carbidic clusters and quasi-free-standing graphene Phys Rev Lett 2009 103 166101 19905709 10.1103/PhysRevLett.103.166101

[B9] Phark S Borme J León Vanegas A Corbetta M Sander D Kirschner J Direct observation of electron confinement in epitaxial graphene nanoislands ACS Nano 2011 5 8162 8166 10.1021/nn2028105 21942619

[B10] Li J Schneider WD Crampin S Berndt R Tunneling spectroscopy of surface state scattering and confinement Surf Sci 1999 422 95 106 10.1016/S0039-6028(98)00890-5

[B11] Kliewer J Berndt R Crampin S Scanning tunneling spectroscopy of electron resonators New J Phys 2001 3 22

[B12] Hämalainen SK Sun Z Boneschanscher MP Uppstu A Ijäs M Harju A Vanmaekelbergh D Liljeroth P Quantum-confined electronic states in atomically well-defined graphene nanostructures Phys Rev Lett 2011 107 236803 22182115 10.1103/PhysRevLett.107.236803

